# Draft genomes and initial characteriaztion of siderophore producing pseudomonads isolated from mine dump and mine drainage

**DOI:** 10.1016/j.btre.2019.e00403

**Published:** 2019-11-23

**Authors:** Marika Hofmann, Thomas Heine, Vivian Schulz, Sarah Hofmann, Dirk Tischler

**Affiliations:** a Institute of Biosciences, Chemistry and Physics Faculty, TU Bergakademie Freiberg, 09599 Freiberg, Germany; b Microbial Biotechnology, Faculty of Biology and Biotechnology, Ruhr-Universität Bochum, 44780 Bochum, Germany

**Keywords:** *Pseudomonas*, Siderophore production, Beech wood hydrolysate, Pyochelin, Pyoverdine, Pseudomonine

## Abstract

Siderophores are of high interest for biotechnological, pharmaceutical, agricultural and industrial applications. Although they are synthesized by various organisms, the yield is usually low which hindrances their suitability for broad range uses. Thus, it is necessary to identify novel producers and to increase the understanding of the biosynthesis pathways. Herein we report the isolation of two novel *Pseudomonas* strains and the identification of the gene clusters for the biosynthesis of pseudomonine as well as pyochelin and pyoverdine.

## Highlights

• High and stable siderophore production.
• Identification of siderophore biosynthesis gene clusters.
• Beech wood hydrolysate as alternative carbon source.

Iron is an essential element for living organisms and involved in numerous cellular processes. Thus, the maintenance of a proper intercellular level is critical [1]. However, iron is not easily bioavailable due to the formation of insoluble forms at aerobic conditions and neutral pH, although it is one of the most abundant elements. One strategy to circumvent this issue is the employment of small carriers called siderophores [2]. These are produced as secondary metabolites by various organisms including prokaryotes (bacteria [3,4], cyanobacteria [5] and archaea [6]) as well as eukaryotes (plants [7], fungi [8] and mammals [9]). Siderophores differ in structure and chemical properties but can be classified according to their metal chelating functional groups into hydroxamate, catecholate, carboxylate and mixed type [10]. The chemical structure also defines their affinity for iron but also other metals. Besides their physiological role, they have been shown to be promising candidates for various industrial, agricultural and medical applications [4,11]. However, the biosynthesis pathways of siderophores are usually complex and the yield is modest. Thus, it is currently laborious and expensive to produce siderophores in sufficient amounts. Therefore, the isolation, identification and characterization of siderophore producers is of high importance to facilitate an application of these compounds. Especially pseudomonads are known to be versatile siderophore producers [12]. In this study, we isolated two novel strains that offer a steady siderophore production rate and especially showed a good affinity for other metals like Al and Cu.

**Fig. 1:**
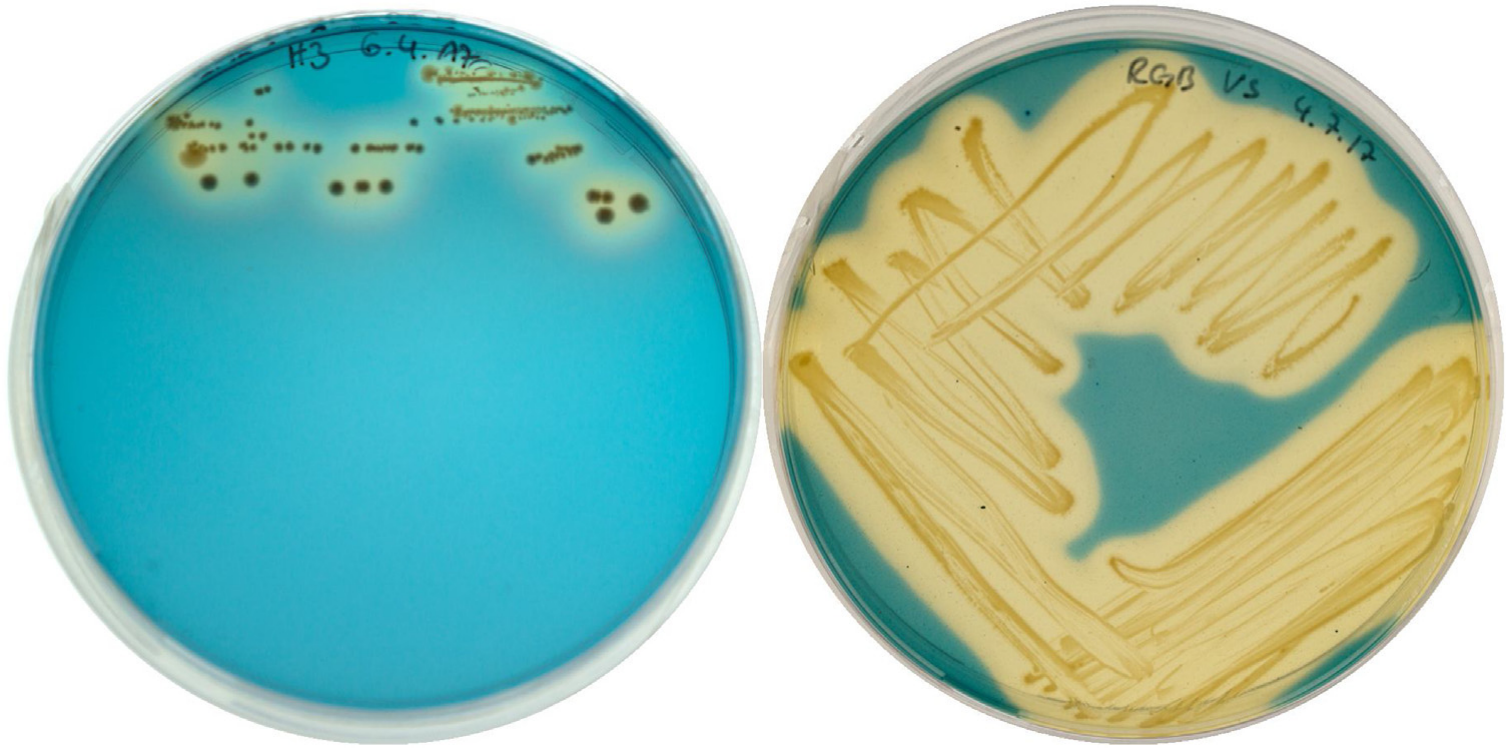
Isolates H3 (left) and RGB (right) plated on CAS-agar selection plates. Production and secretion of siderophores by the bacteria is indicated by formation of yellowish halos around the colonies.

The herein described strains have been isolated from a heap in “Neuhilbersdorf” (strain H3, 50°55’07.1“N 13°22’19.2“E, 2016/12/13) and wet soil next to the mine drainage region “Roter Graben” in Tuttendorf (strain RGB, 50°56’24.1“N 13°22’19.6“E, 2017/01/18). They were selected as potent siderophore producers by evaluation on Chrome azurol S (CAS)-agar plates [13,14] (Fig. 1). Further, they showed to be selective for other metals than iron, making them interesting candidates for further investigations.

Genome sequencing was done to reveal the genus and to gain information about siderophores types that are supposed to be produced. Therefore, both strains were cultivated in 50 ml LB media, harvested after 3 days and DNA was isolated as described previously [15].

**Fig. 2:**
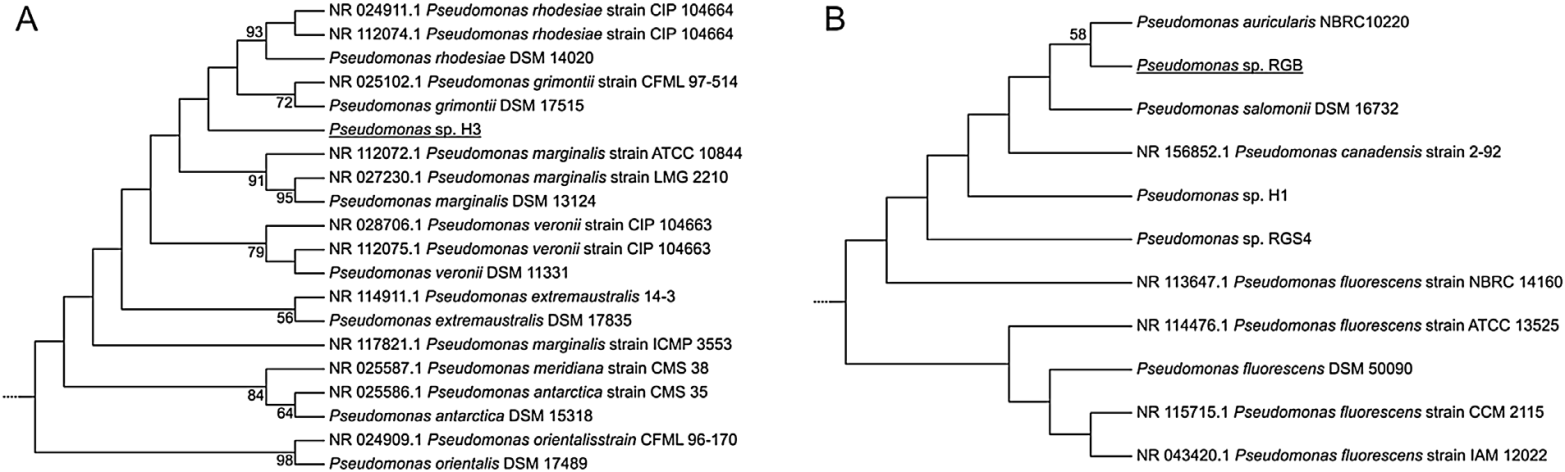
Separated subtrees with focus on related strains of isolate H3 (**A**) and isolate RGB (**B**). Phylogenetic analysis of the isolates H3 and RGB was done based on a 16S rDNA sequences. The complete multiple sequence alignment was prepared with 167 16S rDNAs by applying the ClustalW algorithm. The maximum likelihood tree was constructed by using the MEGAX software and bootstraps of 5,000 replicates [39]. Bootstrap values above 50 % are indicated.

The DNA library preparation, genome sequencing, assembly, annotation and analysis were done as described previously [16]. Therewith a genome coverage of 247x for strain H3 and 249x for strain RGB was obtained, respectively. The results of the genome sequencing are summarized in Table 1. For isolate H3, 49 contigs (49 > = 1000 bp) were identified that cover about 5.8 Mbp with an average G + C content of 58.8 % (N50 = 175970; N75 = 108569; L50 = 11; L75 = 21). For isolate RGB, the assembly resulted in 43 contigs (41 > = 1000 bp) covering about 6.3 Mbp with an average G + C content of 60.5 % (N50 = 359427; N75 = 161348; L50 = 6; L75 = 12). A 16S rDNA-based phylogenetic analysis was done for both isolates revealing that both are classified as *Pseudomonas* species (Fig. 2). Isolate H3 clusters together with *P. rhodesiae* (99.3 % identity to strain DSM 14020) *and P. grimontii* (99.2 % identity to strain DSM 17515). However, strain H3 forms a separate branch and might be related to a novel species, which has to be proven. Isolate RGB is situated close to *P. auricularis* NBRC 10220 (100 % identity to strain NBRC 10220) and is therefore probably associated to this species.

**Table 1:** Genome statistics of the isolated pseudomonads.

Genome Feature	*Pseudomonas* sp. H3	*Pseudomonas* sp. RGB
Sum of contig length (bp)	5,865,301	6,336,192
Contigs	49 (> 3000 bp)	43 (> 950 bp)
G + C content (%)	58.8	60.5
Protein coding genes	5361	5723
Average gene length (bp)	950	960
Coding percentage (%)	87	87
tRNAs	42	44
rRNAs	3	3

**Fig. 3:**
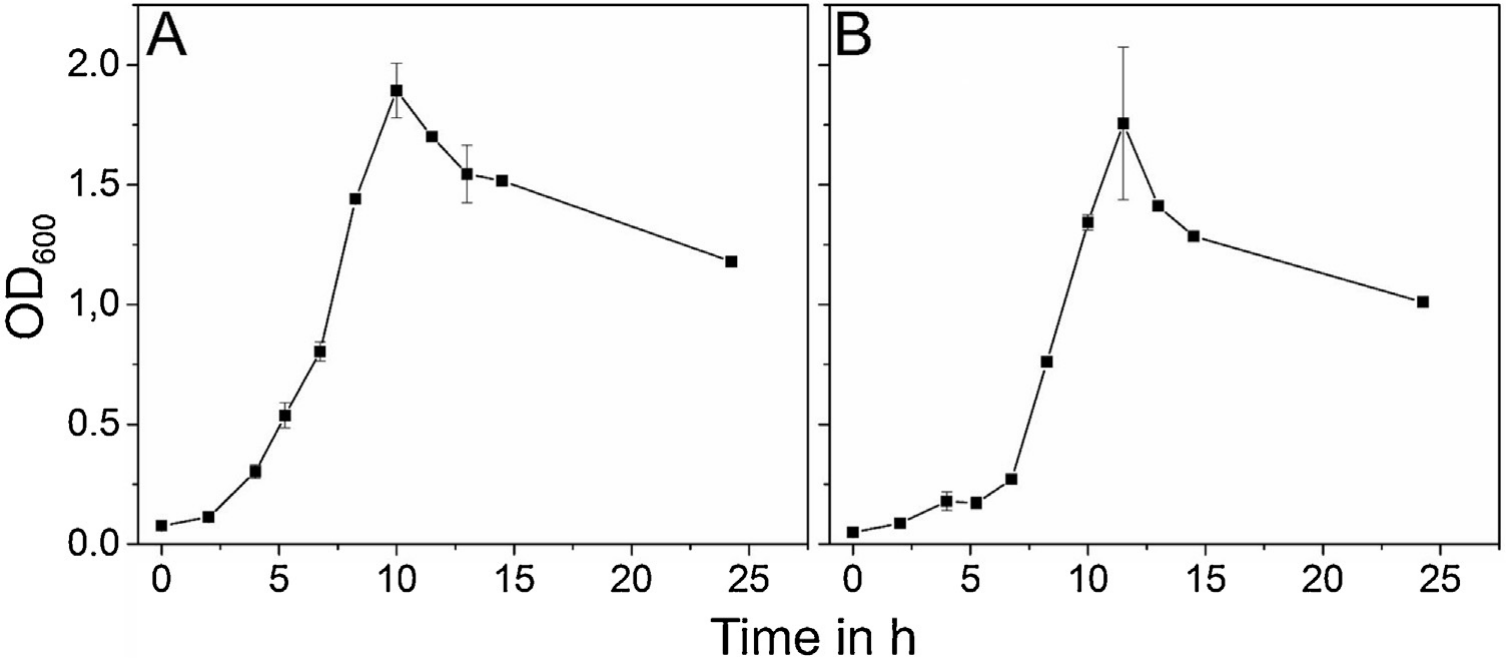
Growth of (A) *Pseudomonas* sp. H3 and (B) *Pseudomonas* sp. RGB on 5 mM beech wood hydrolysate at 30 °C and 160 rpm.

Biochemical typing of both strains was done by Api20 NE (Table 2). Most of the metabolic properties of the isolates resemble those of the reference strains. Both strains are Gram-negative as well as catalase and oxidase positive. Notable differences can be found for isolate H3 in comparison to the nearest relatives on 16S rDNA base. It does not show growth on L-Arabinose and N-acetylglucosamine, while species *rhodesiae* and *grimontii* are able to use these compounds as carbon source. Further, strain H3 did not show fluorescence on Kings B or CSGA medium [17] what indicates that this strain is producing a non-fluorescent siderophore. Strain RGB showed fluorescence only on CSGA media which indicates that this strain might produce fluorescent siderophores. Both strains are able to grow on beech wood hydrolysate (BWH) as cheap alternative to glucose as carbon source for the production of siderophores [18] (Fig. 3). Therefore, both strains were cultivated on minimal media [19] containing 5 mM BWH for 25 h at 30 °C and 160 rpm. The doubling time is with 1.75–2.25 h in a comparable order of magnitude to glucose-grown pseudomonads (Table 3).

**Table 2:** Growth of *Pseudomonas* isolates in comparison to *Pseudomonas* type strains.

	*Pseudomonas* sp. H3	*Pseudomonas* sp. RGB	*P. fluorescens* DSM 50090	*P. fulva* DSM 17717	*P. marginalis* DSM 13124	*P. poae* DSM 14396	*P. simiae* DSM 18861	*P. rhodesiae* DSM 14020	*P. grimontii* DSM 17515*
Growth at 4 °C	+	+	+	+	+	+	+	+	+
Growth at 20 °C	+	+	+	+	+	+	+	+	n.d.
Growth at 37 °C	–	–	–	+	–	–	–	–	n.d.
Fluorescence on Kings B media	–	–	+	–	+	+	+	n.d.	+
Fluorescence on CSGA media	–	+	n.d.	n.d.	n.d.	n.d.	n.d.	n.d.	n.d.
Oxidase test	+	+	+	–	+	+	+	+	+
D-Glucose	+	+	+	+	+	+	+	+	+
L-Arabinose	–	+	+	+	+	+	+	+	+
D-Mannose	+-	+	+	+	+	+	+	+	+
Rhamnose	–	–	–	–	–	n.d.	–	–	+
Trehalose	+	+	+	–	+	+	n.d.	+	+
Succinate	+	+	+	+	n.d.	n.d.	n.d.	n.d.	+
D-Mannitol	+	+	+	–	+	+	+	+	+
N-Acetylglucosamine	–	+-	+	–	+	+	–	+	+
D-Maltose	–	–	–	–	–	–	–	–	–
Potassium gluconate	+	+	+	–	+	+	+	+	+
Decanoic acid	+-	+	+	+	+	+	+	+	n.d.
Adipic acid	–	–	–	–	–	–	–	–	n.d.
Malic acid	+	+	+	–	+	+	+	+	n.d.
Trisodium citrate	+	+	+	+	+	+	+	+	+
Phenylacetate	–	–	–	+	–	–	–	–	–
Benzoate	+	–	+	–	n.d.	n.d.	n.d.	n.d.	+-
Serine	+	+	+	+	n.d.	n.d.	n.d.	n.d.	+
Decane	+	–	–	+	n.d.	n.d.	n.d.	n.d.	n.d.

(+) growth; (−) no growth; (+−) slow growth; (n.d.) not determined; *determined for at least 90 % of the isolated strains.

**Table 3:** Doubling times of *Pseudomonas* isolates and other *Pseudomonas* strains.

Strain	Doubling time in h	Substrate	References
*Pseudomonas* sp. H3	2.25	Beech wood hydrolysate	This study
*Pseudomonas* sp. RGB	1.75	Beech wood hydrolysate	This study
*P. fluorescens*	1.42	Glucose	[20]
*P. putida*	1.67	Glucose	[21]
*P. fragi*	0.83	Glucose	[22]
*P. chlororaphis*	1.25	Glucose	[23]

The biological subsystem distribution of the annotated genes based on RAST can be found in Table 4 [24]. A subsystem coverage of about 50 % was achieved for both isolates. Herein, 1.5 % of the genes of strain H3 and 2.4 % of the genes of strain RGB are supposed to be related to iron acquisition and metabolism, respectively. An additional genome analysis was executed on the antismash 5.0 platform to estimate the production of secondary metabolites and especially siderophores [25]. For *Pseudomonas* sp. H3, twelve secondary metabolite gene clusters were identified whereas four are annotated as putative siderophore gene clusters (cluster 1, 4, 9 and 10; Table 5). Cluster 1 contains several genes that can be found in the pyoverdine biosynthesis cluster, but no relevant peptide synthetase. Thus, it is unlikely that pyoverdines can be produced, especially as strain H3 does not show fluorescence. Cluster 2 and 9 contain IucA/IucC-like synthetases, which indicate the production of hydroxamate siderophores like aerobactin and desferrioxamine. This remains to be proven as these clusters also do not contain related biosynthesis components like an N-hydroxylase or a decarboxylase. At least, manual annotation showed that only cluster 10 contains the gene set for the production of the isoxazolidone siderophore pseudomonine (Table 6) [[26], [27], [28], [29]]. This siderophore was found in *Pseudomonas entomophila* and *Pseudomonas fluorescens* strains [30]. However, not much is known about the biochemical and metal binding properties of this siderophore.

**Table 4:** Biological subsystem distribution of annotated genes in the isolates H3 and RGB.

		*Pseudomonas* sp. H3		*Pseudomonas* sp. RGB	
	Subsystem coverage	52 %		51 %	
Code	Description	Value	Percent	Value	Percent
A	Cofactors, vitamins, prosthetic groups, pigments	317	7.8	348	8.1
B	Cell wall and capsule	214	5.2	180	4.2
C	Virulence, disease and defense	166	4.1	153	3.5
D	Potassium metabolism	30	0.7	27	0.6
E	Miscellaneous	100	2.5	84	1.9
F	Phages, prophages, transposable elements, plasmids	14	0.3	26	0.6
G	Membrane transport	231	5.7	251	5.8
H	Iron acquisition and metabolism	60	1.5	102	2.4
I	RNA metabolism	224	5.5	207	4.8
J	Nucleosides and nucleotides	115	2.8	128	3.0
K	Protein metabolism	279	6.8	289	6.7
L	Cell division and cell cycle	33	0.8	33	0.8
M	Motility and chemotaxis	120	2.9	137	3.2
N	Regulation and cell signaling	126	3.1	139	3.2
O	Secondary metabolism	6	0.1	4	0.1
P	DNA metabolism	110	2.7	120	2.8
Q	Fatty acids, lipids, and isoprenoids	199	4.9	190	4.4
R	Nitrogen metabolism	50	1.2	50	1.2
S	Dormancy and sporulation	5	0.1	3	0.1
T	Respiration	148	3.6	128	3.0
U	Stress response	195	4.8	199	4.6
V	Metabolism of aromatic compounds	101	2.5	130	3.0
W	Amino acids and derivatives	707	17.3	728	16.9
X	Sulfur metabolism	59	1.4	103	2.4
Y	Phosphorus metabolism	45	1.1	79	1.8
Z	Carbohydrates	423	10.4	480	11.1

**Table 5:** Secondary metabolite clusters identified in *Pseudomonas* sp. H3 with antiSMASH5.0.

Cluster	Type	From	To	Most similar known cluster	Similarity	MIBiG BGC-ID
1	Resorcinol	1	32,672	Pyoverdine	12 %	BGC0000413
2	NRPS-like	1	20,451	Mangotoxin	71 %	BGC0000387
3	Hserlactone	60,840	81,502	–	–	–
4	Siderophore	117,806	129,707	–	–	–
5	Arylpolyene	2,428	46,051	APE Vf	40 %	BGC0000837
6	Bacteriocin	16,482	28,126	–	–	–
7	Bacteriocin	50,089	60,982	–	–	–
8	Betalactone	12,457	35,644	Fengycin	13 %	BGC0001095
9	Siderophore	260,231	272,153	–	–	–
10	NRPS	131,179	182,321	Pseudomonine	100 %	BGC0000410
11	Ectoine	225,551	235,937	–	–	–
12	transAT-PKS,NRPS	245,969	338,222	Leinamycin	8 %	BGC0001101

**Table 6:** Pseudomonine related gene cluster in *Pseudomonas* sp. H3.


Putative siderophore: Pseudomonine					
*Pseudomonas* sp. H3		*Pseudomonas fluorescens* WCS374 (GenBank: **EF457930** and **Y09356**)			
Locus	Gene	Description	Accession	% ID	% Coverage
4876	–	Ferrichrome-iron receptor	–	–	–
4877	orf10	Iron compound ABC uptake transporter substrate-binding protein	**ABS50193**	84	76
4878	orf9	Ferric siderophore ABC transporter, ATP-binding protein	**ABS50192**	89	100
4879	orf8	Iron compound ABC uptake transporter permease protein PiuC	**ABS50191**	85	99
4880	orf7	Iron compound ABC transporter, permease protein	**ABS50190**	95	100
4881	orf6	Putative ABC transporter permease/ATP-binding protein	**ABS50189**	87	100
4882	orf5	Putative ABC transporter permease/ATP-binding protein	**ABS50188**	87	100
4883	orf4	Sigma factor, ECF-superfamily	**ABS50187**	81	98
4884	*pmsG*	NRPS	**ABS50186**	79	100
4885	*pmsF*	N-hydroxylase	**ABS50185**	87	73
4886	*hyp*	Hypothetical protein	–	–	–
4887	*pmsD*	NRPS	**ABS50184**	82	100
4888	*pmsC*	Isochorismate synthase (EC 5.4.4.2)	**CAA70528**	86	100
4889	*pmsE*	NRPS	**CAA70529**	90	100
4890	*pmsA*	Pyridoxal-dependent histidine decarboxylase	**CAA70530**	86	97
4891	*pmsB*	Isochorismate pyruvate-lyase (EC 4.-.-.-)	**CAA70531**	76	100

For strain RGB, twelve secondary metabolite gene clusters were identified whereas three are annotated as putative siderophore gene cluster for the production siderophores (cluster 1, 2 and 8; Table 7). A manual analysis on genome level allowed for the identification of siderophore gene clusters contain the relevant genes for production of pyochelin (cluster 2) and pyoverdine (cluster 1 and 8). The annotation of the respective genes can be found in Table 8, Table 9. The pyochelin cluster is complete [31,32]. However, the cluster organization of pyoverdine is different in strain RGB compared to the reference in *Pseudomonas aeruginosa* PA01. Some regulatory genes and the cluster *pvcABCD* (relevant for the biosynthesis of the pyoverdine chromophore) are missing [33]. The fragmentation of the clusters might be a result of the draft genome sequence. However, all required genes that are needed for the biosynthesis and transport of pyoverdine are present in strain RGB [34]. Pyochelin as well as pyoverdines are known to be fluorescent, which harmonizes the observation of strain RGB on CSGA medium.

**Table 7:** Secondary metabolite clusters identified in *Pseudomonas* sp. RGB with antiSMASH5.0.

Cluster	Type	From	To	Most similar known cluster	Similarity	MIBiG BGC-ID
1	NRPS	33,192	100,383	Pyoverdine	10 %	BGC0000413
2	NRPS	48,274	100,804	Pyochelin	100 %	BGC0000412
3	Phenazine,hserlactone	196,591	219,395	Pyocyanine	100 %	BGC0000936
4	Thiopeptide	433,489	463,427	Lipopolysaccharide	5 %	BGC0000774
5	Arylpolyene	304,851	348,426	APE Vf	45 %	BGC0000837
6	NRPS	135,021	186,674	Viscosin	75 %	BGC0001312
7	NAGGN	85,373	100,323	–	–	–
8	NRPS	133,328	186,224	Pyoverdine	10 %	BGC0000413
9	Bacteriocin	593,687	604,532	–	–	–
10	Bacteriocin	417,217	428,095	–	–	–
11	Betalactone	179,482	202,682	Fengycin	13 %	BGC0001095
12	NRPS-like	1	21,957	Mangotoxin	71 %	BGC0000387

**Table 8:** Pyochelin related gene cluster in *Pseudomonas* sp. RGB.


Putative siderophore: Pyochelin					
*Pseudomonas* sp. RGB		*Pseudomonas aeruginosa* PAO1 (GenBank: **X82644** and **AF074705** and **U03161**)			
Locus	Gene	Description	Accession	% ID	% Coverage
3845	*pchA*	Menaquinone-specific isochorismate synthase (EC 5.4.4.2)	**CAA57969**	44	99
3846	*pchB*	Isochorismate pyruvate-lyase (EC 4.2.99.21)	**CAA57968**	61	100
3847	*pchC*	Pyochelin biosynthetic protein PchC, predicted thioesterase	**CAA57967**	62	92
3848	*pchD*	2,3-Dihydroxybenzoate-AMP ligase (EC 2.7.7.58)	**CAA57966**	71	95
3849	*pchR*	Transcriptional regulator PchR	**NP_252917**	71	100
3850	*pchE*	NRPS	**AAC83656**	59	100
3851	*pchF*	NRPS	**AAC83657**	62	100
3852	*pchG*	Thiazolinyl imide reductase	**AAK01463**	61	99
3853	*pchH*	Putative ABC iron siderophore transporter	**AAK01464**	54	99
3854	*pchI*	Putative ABC iron siderophore transporter	**AAK01462**	55	100
3855	*fptA*	Fe(III)-pyochelin outer membrane receptor	**AAC43213**	74	97
3856	*fptB*	Hypothetical protein in pyochelin cluster	**AAC43214**	58	21
3857	*fptC*	Putative iron-regulated membrane protein	**AAC43215**	51	72
3858	*fptX*	inner-membrane permease	**NP_252908**	67	93

**Table 9:** Pyoverdine related gene clusters in *Pseudomonas* sp. RGB.


Putative siderophore: Pyoverdine					
*Pseudomonas* sp. RGB		*Pseudomonas aeruginosa* PA01 (GenBank: **AY765259** and **AE004091**)			
Locus	Gene	Description	Accession	% ID	% Coverage
1153	*pvdP*	Tyrosinase	**AAX16288**	65	96
1154	*pvdM*	Periplasmic enzyme	**AAX16289**	76	99
1155	*pvdN*	Periplasmic enzyme	**AAX16290**	62	100
1156	*pvdO*	Periplasmic enzyme	**AAX16291**	75	93
1157	*pvdF*	N5-hydroxyornithine transformylase	**AAX16292**	80	99
1158	*pvdE*	ABC transporter (secretion)	**AAX16293**	78	100
1159	*fpvA*	Ferripyoverdine receptor protein	**AAX16294**	65	98
1160	*pvdD*	NRPS	**AAX16295**	54	99
1161	*pvdJ*	NRPS	**AAX16296**	48	97
1162	*pvdI*	NRPS	**AAX16297**	56	100
2550	*mbtH*	MbtH-like NRPS chaperone	**AAG05800**	85	95
2551	*pvdH*	Aminotransferase	**AAG05801**	85	97
2560	*pvdL*	NRPS	**AAG05812**	74	100
2561	*pvdG*	Thioesterase	**AAG05813**	53	98
2562	*pvdS*	ECF sigma factor	**AAG05814**	92	93
4143	*pvdQ*	Ntn-type hydrolase	**AAG05773**	54	98
5639	*opmQ*	Outer membrane pyoverdine eflux protein	**AAG05779**	63	97
5640	*pvdT*	Pyoverdine efflux carrier and ATP binding protein	**AAG05778**	81	98
5641	*pvdR*	Pyoverdine specific efflux protein	**AAG05777**	72	99
5642	*fpvI*	ECF sigma factor	**AAG05775**	72	99
5643	*pvdA*	L-ornithine N5-oxygenase	**AAG05774**	75	99

The siderophore production was tested in 5 and 100 ml scale with the glucose as sole carbon source. Therefore, precultures were grown for three days in LB medium (30 °C, 160 rpm), harvested by centrifugation (10,000 × *g*), washed twice with sterile saline, and re-suspended in 10 % of the initial volume with sterile saline. The main culture was inoculated 1:50 with cell suspension. To produce siderophores, a M9 minimal medium with low phosphate content was chosen containing 1.28 g l^−1^ Na_2_HPO_4_, 0.3 g l^−1^ KH_2_PO_4_, 0.5 g l^−1^ NaCl, 1 g l^−1^ NH_4_Cl and 10 ml l^−1^ goodiemix. Goodiemix solution consists of 385 mM MgSO_4_, 10 mM CaCl_2_, 0.1 mM thiamine and 125 ml l^−1^ trace element solution (49 g l^−1^ MgCl_2_, 2 *g* l^−1^ CaCl_2_, 1.44 g l^−1^ ZnSO_4_ · 7 H_2_O, 0.85 g l^−1^ MnSO_4_ ∙ H_2_O, 0.24 g l^−1^ CuSO_4_ · 5 H_2_O, 0.06 g l^−1^ H_3_BO_3_, 51 ml l^−1^ HCl). 20 mM glucose was added as substrate. All glassware used for siderophore production was washed with 6 M HCl in order to remove iron [35]).

**Fig. 4:**
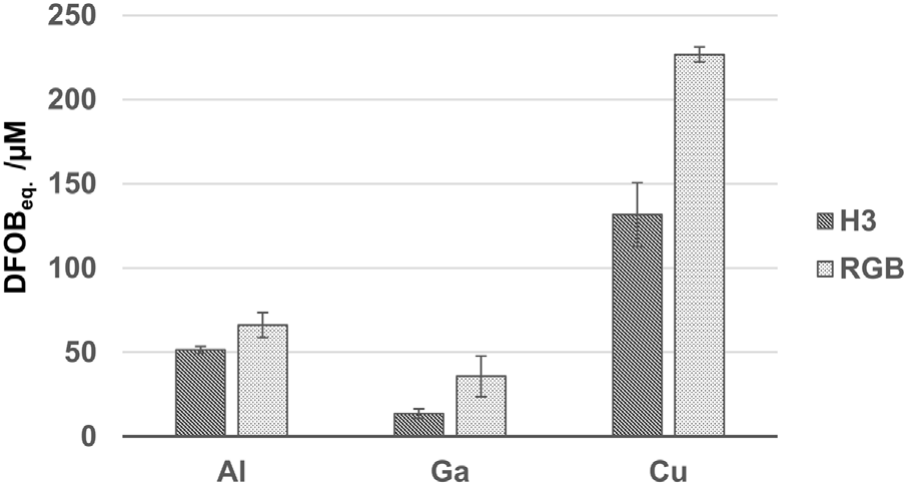
Binding of the metal ions Al^3+^, Ga^3+^ and Cu^2+^ by culture supernatants of *Pseudomonas* sp. H3 and RGB. DFOB_eq._ - desferrioxamine B equivalents.

The highest siderophore production was determined after 3 days with about 130 μM* (* (desferriooxamine B) equivalent [14]) in 5 ml scale. This is in a similar range compared to *P. aeruginosa* strains, although it has to be mentioned that the cultivation conditions are slightly different and the concentration was determined already after 1 day [36,37] (Table 10). Further, it can be seen that the siderophore concentration is lower in a bigger cultivation volume. Binding of other metal ions (Al^3+^, Ga^3+^ and Cu^2+^) was determined in the culture supernatant by adapted CAS-assays (Fig. 4) [14]. All of the tested metal ions can be chelated by the siderophores. This is in accordance with previous findings on pyoverdin and pyochelin [38]. For pseudomonine no other ligands than iron have been tested so far.

**Table 10:** Siderophore production of strain H3 and RGB on glucose in 5 ml and 100 ml scale.

Strain	Scale in ml	Carbon source	Siderophore production in μM	After x days	References
*Pseudomonas* sp. H3	5	20 mM Glucose	135*	3	This study
	100	20 mM Glucose	80*	3	
*Pseudomonas* sp. RGB	5	20 mM Glucose	130*	3	This study
	100	20 mM Glucose	70*	3	
*P. aeruginosa* FP6	100	56 mM Glucose	20	n.s.	[37]
		34 mM Succinate	125	1.5	
*P. aeruginosa* PSS	3500	56 mM Glucose	180	1.25	[36]
		25 mM Succinate	60	1	
		7 mM Glutamic acid	140	1	

n.s. – not specified.

* Calculated in desferrioxamine B equivalents according to [14].

## Nucleotide sequence accession numbers

The genome sequences were deposited at the DDBJ/ENA/GenBank under the accession numbers (*Pseudomonas* sp. H3: VMSG00000000; BioProject: **PRJNA556330**) and (*Pseudomonas* sp. RGB: VMSH00000000; BioProject: **PRJNA556336**).

## Author contribution

MH isolated the strains. MH, VS and SH performed the strain characterization including growth experiments and siderophore production. MH and TH performed the DNA isolation, genome sequencing, annotation and analyzes. TH, MH and DT wrote the manuscript.

## Declaration of Competing Interest

The authors declare that they have no known competing financial interests or personal relationships that could have appeared to influence the work reported in this paper.
